# The association between microaggressions and mental health among UK trans people: a cross-sectional study

**DOI:** 10.1007/s00127-024-02775-2

**Published:** 2024-09-26

**Authors:** Talen Wright, Gemma Lewis, Talya Greene, Ruth Pearce, Alexandra Pitman

**Affiliations:** 1 Division of Psychiatry, Faculty of Brain Sciences, University College London, London, UK; 2 Clinical, Educational and Health Psychology, University College London, London, UK; 3 School of Education, University of Glasgow, Glasgow, UK; 4 Center for Applied Transgender Studies, Chicago, IL USA; 5 Camden and Islington NHS Foundation Trust, London, UK

**Keywords:** Transgender, Microaggressions, Depression, Anxiety, Suicidal ideation, Suicide attempt

## Abstract

**Purpose:**

Epidemiological studies investigating the mental health impacts of microaggressions in the trans population have tended to have methodological limitations, including a lack of validated measures, raising concerns about the validity of their findings. To address this evidence gap, we investigated the associations between microaggressions and poor mental health (depression; anxiety; non-suicidal self-harm [NSSH]; suicidal thoughts; suicide attempt) amongst trans people.

**Methods:**

We conducted a cross-sectional survey of 787 trans adults in the UK, measuring mental health and exposure to microaggressions using the Gender Identity Microaggressions Scale (GIMS). Using univariable and multivariable linear and logistic regression models we tested for an association of microaggressions with depressive symptoms (PHQ-9), anxiety symptoms (GAD-7), lifetime NSSH, lifetime suicidal thoughts, and lifetime suicide attempt.

**Results:**

Of the 787 participants, 574 (73%) provided complete data. Microaggressions were a common experience, affecting 97.6% of participants over their lifetime. In adjusted analyses, using sociodemographic and clinical variables, increased microaggression scores were associated with increased depressive symptoms (adjusted coefficient: 1.86 (95%CI = 1.35 to 2.36)), anxiety symptoms (adjusted coefficient: 1.57 (95%CI = 1.09 –2.05)) and with increased odds of NSSH (Odds Ratio [OR]_*adj*_ 1.83 (95%CI = 1.45 –2.30)), suicidal thoughts (OR_adj_ 2.18, (95%CI = 1.52 –3.13)), and suicide attempt (OR_adj_, 1.59, (95%CI = 1.32 –1.92)). In exploratory analyses different GIMS subscales were associated with these various outcomes.

**Conclusions:**

There was evidence of associations between microaggressions and adverse mental health outcomes, as well as to support specific microaggressions being associated with specific outcomes, emphasizing the importance of public health interventions that target microaggressions directed at trans adults. Longitudinal studies are needed to investigate the temporality of the associations between microaggressions and mental health outcomes.

**Supplementary Information:**

The online version contains supplementary material available at 10.1007/s00127-024-02775-2.

## Introduction

Transgender, non-binary, and/or or gender-diverse people (hereafter, trans people) are individuals whose gender identity differs to their sex assigned or registered at birth [1, 2]. Recent census data estimates that this group comprises 0.5% of the total population of England and Wales [3] and 0.44% of the population of Scotland [4]. Evidence from observational studies suggests that when comparing trans to cis people (people who are not trans), trans people have an increased risk of developing anxiety symptoms, depressive symptoms, non-suicidal self-harm (NSSH), suicidal ideation, and suicide attempt [5–10]. Microaggressions are defined as brief commonplace daily verbal, behavioural, or environmental indignities, whether intentional or unintentional, that communicate hostile, derogatory, or negative slights and insults towards marginalised communities [11, 12]. The first use of microaggressions in psychological literature was to describe experiences of ethnic minorities [11, 12]. Currently, there is strong evidence that minority stress plays a role in the mental health outcomes of trans people, which includes acts of discrimination, vicitimisation, structural cissexism and microaggressions [13, 14]. Structural cissexism refers to the systemic and institutionalisation of privilege towards cis people, as distinct from cisnormativity, describing the implicit assumption is that everyone is, or should be, cis, giving rise to biases against those who are trans or gender non-conforming. [15]

There is a paucity of research that investigates the mental health impacts of microaggressions among trans people. The largest study on microaggressions using validated measures recruited 292 participants, finding positive associations between microaggressions and feelings of shame and internalised transnegativity in a small US sample, and a negative association with mental wellbeing [16]. Transnegativity refers to discomfort with one’s own trans identity as a result of internalizing societal attitudes towards trans people [17]. However, this study did not examine mental health outcomes, leading to further questions about the public mental health impact of microaggressions on the trans population. Only one study was found which has recently examined gender identity based microaggressions amongst trans people [18]. The daily diary study found specific associations between microaggressions and mental health outcomes, namely greater frequency of denial of gender identity and depressive symptoms, and greater frequency of denial of societal transphobia with anxiety symptoms [18].

There are further questions around how specific microaggressions influence specific mental health outcomes. Qualitative evidence suggests that specific microaggression experiences may have different emotional, cognitive, and behavioural consequences among trans people [19, 20]. For example, experiences of microaggression that involve being avoided by friends/family/partners can elicit feelings of betrayal among trans people, whilst invasive questions about trans bodies can elicit feelings of invalidation [19]. Given these examples, it is plausible that specific relationships exist between individual microaggression experiences and mental health outcomes, yet no quantitative study has investigated these specific relationships. The aims of this study were to:

1. Investigate the association between gender identity microaggressions and depressive symptoms, anxiety symptoms, NSSH, suicidal thoughts, and suicide attempts amongst trans people.
2. Explore the strength of associations between specific microaggression experiences and depressive symptoms, anxiety symptoms, NSSH, suicidal thoughts, and suicide attempts.

## Methods

### Study design and participants

We conducted a cross-sectional survey of trans people in the United Kingdom (UK), the TRANS: Microaggressions & Mental Health survey. We invited participation from people who identified as trans, non-binary, and/or gender diverse, were aged eighteen years or older and had resided in the UK for 12 months or longer. We used recruitment strategies through social media advertising (Twitter, Facebook, and Instagram), with support from large trans charities and organisations such as Gendered Intelligence sharing survey details. The survey was delivered via Opinio, a web-based survey tool; launched in September 2021, continuing until September 2022. Participants provided written consent via the online survey programme.

### Survey instrument

Variables were selected to include key measures relevant to our hypothesis as well as other social and psychological factors hypothesised to influence the mental health of trans people, as identified in discussion in trans community members.

The survey questionnaire was co-produced with a group of trans people with lived experience of microaggressions and mental health problems, in line with recommended coproduction practices. [21] The aim of the co-production was to improve the acceptability of survey questions and comprehensiveness of the survey whilst also considering overall question burden on participants. The co-production group was comprised of five volunteers, recruited through the first author’s connections with academics and activists within trans communities. All five members were white, under the age of 35, and had experiences of depression, anxiety, suicidal ideation, and suicide attempt, as well as experiences of transphobic microaggressions. Due to funding limitations, co-production team members were not paid for their participation. They were presented with the questionnaire draft and asked to reflect on the acceptability of the survey tool, offer edits for clarification, and comment on the importance of the research questions. We piloted the instrument with the co-production group to ensure the survey programme worked as intended, for example that branching occurred appropriately, and instructions given within the survey were clear.

### Outcomes

Our five main outcomes were:

***Depression symptoms*:** measured using the Patient Health Questionnaire – 9 item version (PHQ-9). The PHQ-9 assesses the severity of depression symptoms, with scores ranging from 0 to 27. The PHQ has good psychometric properties [22], and good convergent validity and internal consistency [23].

***Anxiety symptoms*:** measured using the Generalised Anxiety Disorder Scale – 7 item version (GAD-7). The GAD-7 assesses the severity of anxiety symptoms, with possible total scores ranging from 0 to 21. The GAD-7 has been well validated as a brief screening measure that is sensitive to change and acute symptom presentation [24].

***Lifetime history of non-suicidal self-harm (NSSH), suicidal thoughts, and suicide attempt*:** measured using self-report measures from the Adult Psychiatric Morbidity Survey (APMS) [25]. We created a binary measure denoting the presence or absence of each.

### Exposure

**Microaggressions**: measured using the Gender Identity Microaggression Scale (GIMS). The GIMS is a 14-item scale with five subscales assessing lifetime occurrence of a) denial of gender identity, e.g., refusing to recognise trans people’s gender identity b) misuse of pronouns, e.g., consciously, or unconsciously, referring to a trans person with the wrong pronoun (he, she, and/or they for example) c) invasion of bodily privacy, e.g., asking inappropriate questions about a trans person’s genital configuration d) behavioural discomfort from others, e.g., acting in an uncomfortable manner with a trans person in any setting, and e) denial of societal transphobia, e.g., telling a trans person that experiences of transphobia (such as losing or being refused a job because they are trans) do not exist (see supplementary Table 1 for full list of items). Scores on the scale are summed to produce a total overall score, with higher scores indicating more experiences of gender identity microaggressions over the lifetime. The total scale ranges from 14 to 70 points. The scale has good internal consistency [26]. The GIMS has been used to examine mental health outcomes in the UK trans population, with a small sample of 39 trans people, finding associations between microaggressions and mental health outcomes over a 10 day period [18]

## Confounders

The following confounders were selected based on published literature.

### Perceived gender

Perceived gender in this context describes the participants’ beliefs about how others perceive their gender. Trans people frequently report distress when other people’s perceptions of their gender conflicts with their own. Being visibly trans opens potential negativity from others, especially in transphobic and trans-hostile societies and communities. Conversely, being perceived to be the gender one identifies with may offer some protection against trans-hostility, especially in the case of binary presenting trans people (e.g. those who present as women being identified as women).

Individuals who believe they are more frequently seen as a trans person, or as the sex they were assigned at birth, experience more microaggressive events [27]. Furthermore, believing one is being perceived as trans or as the sex a trans person was assigned at birth has been linked to increased depressive symptoms [28].

### Age

Age has been identified as a factor associated with microaggressions, with a tendency for the experience of microaggressions to decrease as age increases [27]. A USA-based study involving 223 trans individuals found that those aged 25–34 had a mean score of 0.75 points lower on microaggressions compared to those aged 18–35 [29]. Similarly, individuals aged 35–49 scored a mean of 1.32 points lower than those aged 18–24. Additionally, evidence suggests that age is associated with depressive symptoms, anxiety symptoms, NSSH, suicidal thoughts, and suicide attempts [30–33]. Population-based studies in the UK indicate that individuals aged 45–59 have a significantly higher prevalence of probable depressive disorder compared to those aged 16–29 [34]. Similarly, there are age-related associations with anxiety symptoms and NSSH, suicidal thoughts, and suicide attempts, with a decrease in suicidal thoughts, plans, and attempts as age increases [33]. Moreover, anxiety symptoms tend to be higher in younger age groups compared to older age groups [35]. Given these findings, it is plausible that age may act as a confounding factor in the relationship between microaggressions and depressive symptoms within our sample. We asked participants to choose an age category from the following: 18–24, 25–34, 35–44, 45–54, 55–64, 65 + .

### Education

Education was selected as an indicator of socioeconomic status (SES). SES is defined as a person’s work experience and of an individual’s economic access to resources and social position in relation to others [36]. There is evidence that education level may predict microaggressions [37] and that education is also a predictor of mental health distress [37]. Participants were asked to report their highest educational attainment with the following options: No qualifications, GSCEs or equivalent, A-levels/Scottish Highers, University Degree (e.g., BSc, BA), Master’s Degree or equivalence, Doctorate (e.g., MD or PhD), or Vocational Qualifications.

### Sexuality

Lesbian, Gay, and Bisexual (LGB) people are at a higher risk of adverse mental health conditions such as depressive symptoms, anxiety symptoms, and suicidal thoughts and behaviours when compared to heterosexual people [38, 39]. There is also evidence that LGB individuals experience specific forms of microaggressions relating to their sexuality [40, 41]. These interpersonal and environmental microaggressions are manifested in the form of hostile messages towards sexual minorities [42]. There is evidence that LGB people are at increased risk of microaggressions [43] and that these microaggressions may increase risk of depressive symptoms and anxiety symptoms [43]. Participants were able to select or self-define their sexuality. For the purposes of analysis, these were combined into a binary variable with the two categories representing LGB/Queer/Questioning/Asexual/Aromantic, and heterosexual.

### Disability

We selected disability as a confounder because people with disabilities, including physical disability and neurodiversity, are at an increased risk of experiencing disability-specific microaggressions [44]. Examples of microaggressions specific to those with disabilities include patronisation, desexualisation, and second-class citizenship [41]. People with disabilities also show an increased risk of developing poor mental health when compared to able-bodied people [45, 46]. People with disabilities who experienced ableist microaggressions had higher scores on depressive symptoms and anxiety symptoms measures compared to those who did not experience ableist microaggressions [47]. Participants were asked to report whether they had a disability lasting 12 months or longer, and to indicate whether this reduced their ability to carry out day to day with the following option, “Not at all”, “Yes a little”, and “Yes a lot”.

### Ethnicity

There is evidence that supports a relationship between membership of ethnic minority communities and experiences of microaggressions [37, 48]. Furthermore, there is evidence supporting a relationship between ethnicity and mental health distress, with ethnicity/race-related stressors increasing susceptibility to depressive symptoms, anxiety symptoms, NSSH, suicidal thoughts, and suicide attempt [49]. Ethnicity questions were derived from the Office for National Statistics Census and presented alphabetically [50].

### Stage of physical and/or social transition

Physical transition was taken from the Trans Mental Health Study (2012) where participants are asked to state at which point they are currently on their physical transition. Participants can answer in the following ways: “*No, I have not undergone or propose to undergo any part of a process*”, “*Yes, I have undergone a process*”, “*Yes, I am currently undergoing a process*”, “*Yes, I am proposing to undergo a process*”, and “*Unsure*”, “*Prefer not to say*”, and “*Other*”. Stage of social transition was a separate question and utilised the same response format.

### Primary analysis

**Descriptive statistics:** We described the characteristics of our complete case analytic sample (i.e., those who provided complete data on exposure, confounders, and all five outcomes). We reported characteristics using means and standard deviations, and medians and inter-quartile ranges, as appropriate. To aid interpretation, we reported these based on a median split of the GIMS, describing differences in the sample characteristics and potential confounders between those who experienced high and low levels of microaggressions.

**Regression analyses**: We transformed the GIMS exposure variable into standard deviation units as the unit increase was small relative to the range, to aid interpretation [51]. We used linear regression models with depressive symptoms (PHQ-9) and anxiety symptoms (GAD-7) scores as separate continuous outcomes and the transformed microaggressions scale (GIMS) as a continuous exposure. We used logistic regression models with NSSH, suicidal thoughts, and suicide attempt as separate binary outcomes and the transformed microaggressions scale as a continuous exposure. All analyses were reported before and after adjustment.

## Exploratory analyses

To investigate the possibility that specific microaggression experiences are associated with specific mental health outcomes we first assessed correlations between GIMS subscales using pairwise correlations. We then investigated the associations of each GIMS subscale with outcomes in separate univariable models. We then performed multivariable linear (for depressive symptoms and anxiety symptoms) and multivariable logistic (for NSSH, suicidal thoughts, and suicide attempts) regression models to mutually adjust all five GIMS subscales (but not other confounders). In the final models, we mutually adjusted all GIMS subscales together with confounders in multivariable linear and logistic regression models (respectively) to explore potential associations of the subscales with the mental health outcomes.

### Sensitivity analyses

**Imputation:** To assess the potential influence of missing data when modelling the association between microaggressions and our five outcomes we investigated differences between participants with complete data and those with missing data[52]. Auxiliary variables were selected if they were associated with the exposure and outcomes and were not included in the analysis. The role of the variables was to improve imputation estimates and reduce coefficient bias in the multiple imputation [53] Auxiliary variables were loneliness, and whether the participant was living in their affirmed gender. We used Multiple Imputation by Chained Equations (MICE) to impute twenty-five datasets, which were then combined using Rubin’s rules. We imputed on the exposure (GIMS), outcomes, and confounders. In our analyses we first restricted the sample to those with complete cases on the exposure. Next, we used the imputed data on the exposure, outcomes, and confounders, and restricted to complete case of exposure.

**Loneliness as a putative mediator**: After discussing our initial findings, we decided to explore the potential role of loneliness in our models. We therefore added a post hoc sensitivity analysis to assess whether poor social support might help explain the association between microaggressions and our mental health outcomes, based on the possibility that this might lie on the causal pathway from microaggressions to mental distress. This was based on our theory that experiencing more frequent microaggressions might lead to low perceived social connectedness, which in turn would worsen mental health. We used loneliness as a proxy for poor social support, measured using the 3-item UCLA Loneliness Scale, a validated measure capturing the subjective experience of loneliness [54]. To test for evidence of a potential mediating role, we added loneliness to our final adjusted models and compared the coefficients from these sensitivity analyses to those in our main analyses.

All analyses were conducted using Stata version 17.1 [55].

## Results

### Sample characteristics:

**Fig. 1:**
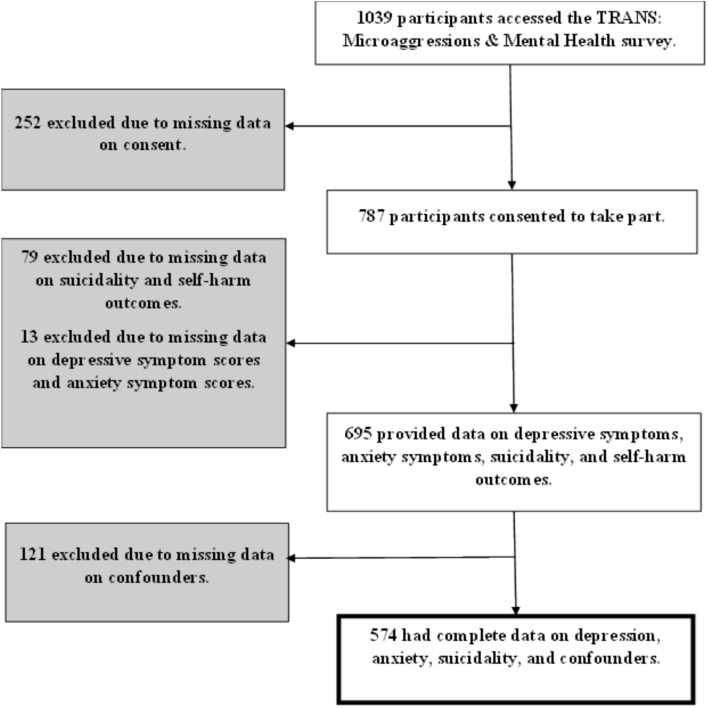
Flow Diagram for participants in the TRANS: Microaggressions & Mental Health baseline study

A total of 787 participants responded to the online advertisement about the TRANS: Microaggressions & Mental Health Survey and took part in the cross-sectional study, of whom 574 (79%) provided complete data on exposure, outcomes, and confounders, comprising our complete case analytical sample (Fig. 1).

Most participants were under the age of 35 (n = 428, 73%), white (n = 525, 91.5%), and resided in England (n = 483, 84%). The mean GIMS score was 42.46 (SD 13.28), and 97.6% (n = 560) endorsed at least one form of lifetime microaggression experience. The most common specific microaggression experience was misuse of pronouns (n = 544, 94.8%), and the least endorsed was behavioural discomfort from others (n = 389, 67.8%).

After splitting participants into those with a high versus low number of microaggression experiences, based on a median split of the GIMS (median = 42; IQR = 33– 52; see Table 1), those who experienced high microaggressions had a mean score of 13.52 (SD 6.46) on the PHQ-9 and a mean score of 11.49 (SD 5.90) on the GAD-7. The high microaggression (HM) group, compared with the low microaggression (LM) group, had a significantly higher proportion of those with a lifetime history of NSSH (HM: 86.6% vs LM: 68.3%), suicidal thoughts (HM: 95.4% vs LM: 86.9%), and suicide attempts (HM: 49.0% vs LM: 30.2%).

**Table 1:** Socio-demographic and clinical characteristics of study participants

Sociodemographic variable	Overall sample (n = 574; 100%)	†Low microaggressions group (n = 268; 100%)	High microaggressions group (n = 306; 100%)
Gender			
Trans women	188 (32.8%)	81 (31.5%)	107 (35.0%)
Trans men	104 (18.1%)	49 (19.1%)	55 (18.0%)
Non-binary	251 (43.7%)	127 (49.4%)	124 (40.5%)
Missing	31 (5.4%)	11 (4.1%)	20 (6.5%)
Currently living in affirmed gender			
No, not living in affirmed gender	98 (17.1%)	56 (20.9%)	42 (13.7%)
Yes, either all or most of the time	476 (82.9%)	212 (79.1%)	264 (86.3%)
††Perceived gender by others			
As a trans person	114 (19.9%)	38 (14.2%)	76 (24.8%)
As the gender identified	116 (20.2%)	53 (19.8%)	63 (20.6%)
As the sex assigned at birth	247 (43.0%)	133 (49.6%)	114 (37.3%)
Does not know	49 (8.5%)	27 (10.1%)	22 (7.2%)
Other	48 (8.4%)	17 (6.3%)	31 (10.1%)
††Physical transition			
No, has not undergone/not relevant	70 (12.2%)	46 (17.2%)	24 (7.8%)
Yes, proposing to undergo	123 (21.4%)	58 (21.6%)	65 (21.2%)
Yes, currently undergoing	200 (34.8%)	71 (26.5%)	129 (42.2%)
Yes, undergone	113 (19.7%)	52 (19.4%)	61 (19.9%)
Unsure/Prefer not to say/Other	68 (11.9%)	41 (15.3%)	27 (8.8%)
††Social transition			
No, has not undergone/not relevant	13 (2.3%)	9 (3.4%)	< 5 (1.3%)
Yes, proposing to undergo	44 (7.7%)	26 (9.7%)	18 (5.9%)
Yes, currently undergoing	161 (28.1%)	81 (30.2%)	80 (26.1%)
Yes, undergone	335 (58.4%)	135 (50.4%)	200 (65.4%)
Unsure/Prefer not to say/Other	21 (3.7%)	17 (6.3%)	< 5 (1.3%)
††Age			
18 to 25	225 (39.2%)	97 (36.2%)	128 (41.8%)
26 to 34	193 (33.6%)	92 (34.3%)	101 (33.0%)
35 to 44	87 (15.2%)	44 (16.4%)	43 (14.1%)
45 to 75 +	69 (12.0%)	35 (13.1%)	34 (11.1%)
††Ethnicity			
Ethnic minority	49 (8.5%)	25 (9.3%)	24 (7.8%)
White	525 (91.5%)	243 (90.7%)	282 (92.2%)
††Education			
No education	11 (1.9%)	< 5 (1.5%)	7 (2.3%)
GCSEs or equivalent	35 (6.1%)	14 (5.2%)	21 (6.9%)
A level(s), Scottish Highers or equivalent	115 (20.0%)	49 (18.3%)	66 (21.6%)
University Degree	193 (33.6%)	91 (34.0%)	102 (33.3%)
Master’s Degree	135 (23.5%)	66 (24.6%)	69 (22.6%)
Doctorate	39 (6.8%)	20 (7.5%)	19 (6.2%)
Vocational Qualifications	46 (8.0%)	24 (9.0%)	22 (7.2%)
Employment			
Unemployed and unable to work	56 (9.8%)	20 (7.5%)	36 (11.8%)
Unemployed and looking for work	40 (7.0%)	28 (10.5%)	12 (3.9%)
Employed, part time	66 (11.5%)	28 (10.5%)	38 (12.4%)
Employed, full time	282 (49.1%)	131 (48.9%)	151 (49.4%)
Student	96 (16.7%)	41 (15.3%)	55 (18.0%)
Full time homemaker/Carer	13 (2.3%)	8 (3.0%)	5 (1.6%)
Country currently residing in			
England	483 (84.2%)	224 (83.6%)	259 (84.6%)
Northern Ireland	6 (1.1%)	< 5 (1.5%)	< 5 (0.7%)
Scotland	66 (11.5%)	31 (11.6%)	35 (11.4%)
Wales	19 (3.3%)	9 (3.4%)	10 (3.3%)
National identity			
British	218 (38.0%)	85 (31.7%)	133 (43.5%)
English	242 (42.2%)	124 (46.3%)	118 (38.6%)
Northern Irish	5 (0.9%)	< 5 (1.1%)	< 5 (0.7%)
Scottish	52 (9.1%)	23 (8.6%)	29 (9.5%)
Welsh	21 (3.7%)	12 (4.5%)	9 (2.9%)
Other	36 (6.3%)	21 (7.8%)	15 (4.9%
Urbanicity			
Urban	423 (73.7%)	200 (74.6%)	223 (72.9%)
Rural	133 (23.2%)	59 (22.0%)	74 (24.2%)
Do not know/Other	18 (3.1%)	9 (3.4%)	9 (2.9%)
Religion/Spirituality			
Buddhist	7 (1.2%)	5 (1.9%)	< 5 (0.7%)
Christian (all denominations)	34 (5.9%)	16 (6.0%)	18 (5.9%)
Hindu	< 5 (< 0.9%)	< 5 (< 1.9%)	< 5 (0.3%)
Jewish	8 (1.4%)	< 5 (< 1.9%)	6 (2.0%)
Muslim	5 (0.9%)	-	5 (1.6%)
Pagan	50 (8.7%)	25 (9.3%)	25 (8.2%)
No religion and/or spiritual beliefs	426 (74.2%)	198 (73.9%)	228 (74.5%)
Any other religion	42 (7.3%)	21 (7.8%)	21 (6.9%)
†† Sexuality			
Asexual	32 (5.6%)	20 (7.5%)	12 (3.9%)
Bisexual	185 (32.2%)	87 (32.5%)	98 (32.0%)
Gay	78 (13.6%)	32 (11.9%)	46 (15.0%)
Heterosexual	26 (4.5%)	16 (6.0%)	10 (3.3%)
Lesbian	75 (13.1%)	38 (14.2%)	37 (12.1%)
Pansexual	72 (12.5%)	27 (10.1%)	45 (14.7%)
Queer	84 (14.6%)	37 (13.8%)	47 (15.4%)
Questioning	11 (1.9%)	6 (2.2%)	5 (1.6%)
Ever experienced denial of gender identity			
No	70 (12.2%)	68 (25.4%)	< 5 (0.7%)
Yes	504 (87.8%)	200 (74.6%)	304 (99.3%)
Ever experienced misuse of pronouns			
No	30 (5.2%)	30 (11.2%)	0 (0.0%)
Yes	544 (94.8%)	238 (88.8%)	306 (100.0%)
Ever experienced invasion of bodily privacy			
No	79 (13.8%)	73 (27.2%)	6 (2.0%)
Yes	495 (86.2%)	195 (72.8%)	300 (98.0%)
Ever experienced behavioural discomfort from others in any setting			
No	185 (32.2%)	154 57.5%	31 (10.1%)
Yes	389 (67.8%)	114 (42.5%)	275 (89.9%)
Ever experienced denial of societal transphobia			
No	116 (20.2%)	108 (40.3%)	8 (2.6%)
Yes	458 (79.8%)	160 (59.7%)	298 (97.4%)
Clinical Characteristics	Overall sample mean (SD), or n (%)	Low microaggressions mean (SD), or n (%)	High microaggressions mean (SD), or n (%)
PHQ-9 (depressive symptoms, past two weeks)	12.06 (6.49)	10.41 (6.13)	13.52 (6.46)
GAD-7 (anxiety symptoms, past two weeks)	10.07 (6.02)	8.46 (5.76)	11.49 (5.90)
††Disability (physical or mental health condition that lasts 12 months or more)			
No	138 (24.0%)	81 (30.2%)	57 (18.6%)
Yes	436 (76.0%)	187 (69.8%)	249 (81.4%)
Disability reducing ability to carry out day to day activities			
Not at all	7 (6.7%)	11 (5.9%)	15 (6.0%)
Yes, a little	69 (66.4%)	128 (68.5%)	145 (58.2%)
Yes, a lot	28 (26.9%)	48 (25.7%)	89 (35.7%)
Ever been diagnosed with anxiety or depressive condition, or drug or alcohol problem			
No	26 (4.5%)		
Yes	511 (89.0%)		
Do not know/Prefer not to say	37 (6.5%)		
Lifetime history of suicidal thoughts			
No	49 (8.5%)	35 (13.1%)	14 (4.63%)
Yes	525 (91.5%)	233 (86.9%)	292 (95.4%)
Lifetime history of suicide attempts			
No	343 (59.8%)	187 (69.8%)	156 (51.0%)
Yes	231 (40.2%)	81 (30.2%)	150 (49.0%)
Lifetime history of non-suicidal self-harm			
No	126 (21.9%)	85 (31.7%)	41 (13.4%)
Yes	448 (78.1%)	183 (68.3%)	265 (86.6%)

Total percentages are given per column

*GCSEs* General certificate of secondary education

*PHQ-9* Patient health questionnaire – 9 item version

*GAD-7* Generalised anxiety disorder scale – 7 item version

†Low versus high microaggressions derived on basis of median split of the GIMS total scale

††Variables selected as confounders in the main analytical models

### Association between total microaggressions score and mental health outcomes.

*Depressive symptoms***:** We found evidence of an association between microaggressions and depressive symptoms, whereby as microaggression experiences increased by one standard deviation (13.28-points) of the GIMS scale, this was associated with an increase in depressive symptom scores (unadjusted coefficient: 2.09, 95%CI = 1.59– 2.60; adjusted coefficient: 1.86, 95%CI = 1.35 –2.36; see Table 2).

**Table 2:** Associations between microaggressions (total GIMS score) and depressive symptoms, anxiety symptoms, suicidality, and non-suicidal self-harm

	*Unadjusted*			Adjusted*		
	Model N	Coefficient (95%CI)	P-value	Model N	Coefficient (95%CI)	P-value
*PHQ-9 – Depressive symptoms* *Past two weeks*	574	2.09 (1.59 to 2.60)	< 0.001	574	1.86 (1.35 to 2.36)	< 0.001
*GAD-7 – Anxiety symptoms* *Past two weeks*	574	1.76 (1.28 to 2.23)	< 0.001	574	1.57 (1.09 to 2.05)	< 0.001

	Model N	OR_crude_ (95%CI)	P-value	Model N	OR_adj_ (95%CI)	P-value
Lifetime suicidal thoughts						
No	574	12.59 (1.85 to 3.62)	< 0.001	574	12.18 (1.52 to 3.13)	< 0.001
Yes						
Lifetime suicide attempt						
No	574	11.66 (1.39 to 1.99)	< 0.001	574	11.59 (1.32 to 1.92)	< 0.001
Yes						
Lifetime non-suicidal self-harm						
No	574	1 1.95 (1.57 to 2.42)	< 0.001	574	11.83 (1.45 to 2.30)	< 0.001
Yes						

*OR*_*crude*_ Unadjusted odds ratio

*OR*_*adj*_ Adjusted odds ratio

*PHQ* Patient health questionnaire

*GAD* Generalised anxiety disorder scale

*GIMS* Gender identity microaggressions scale

^*^ Adjusted for age, perceived gender, ethnicity, sexuality, disability (physical or mental health condition that lasts 12 months or more), education, stage of physical/medical transition, and stage of social transition

*Anxiety symptoms***:** We found evidence of an association between microaggressions and anxiety symptoms, whereby when scores on microaggression experiences increased by one standard deviation of the GIMS scale, this was associated with an increase in anxiety symptom scores (unadjusted coefficient: 1.76, 95%CI = 1.28–2.23; adjusted coefficient: 1.57, 95%CI = 1.09–2.05).

*Lifetime NSSH, suicidal thoughts, and suicide attempt*: We found evidence of an association between microaggression experiences and lifetime NSSH (OR_crude_ 1.95, 95% CI = 1.57–2.42; OR_adj_ 1.83, 95%CI = 1.45 –2.30), lifetime suicidal thoughts (odds ratio [OR]_crude_ 2.59, 95%CI = 1.85–3.62; OR_adj_ 2.18, 95%CI = 1.52–3.13), and lifetime suicide attempt (OR_crude_ 1.66 95%CI = 1.39–1.99; OR_adj_, 1.59, 95%CI = 1.32–1.92).

### Association between specific GIMS subscales and specific mental health outcomes

Correlation coefficient estimates for GIMS subscales ranged from 0.31 to 0.56 (see Table 3), suggesting weak to moderate correlations between each subscale. This justified use of the subscales as exposures in multivariable models.

**Table 3:** Correlation matrix for GIMS subscales

	***1***	***2***	***3***	***4***	***5***
*1. Denial of gender identity*	***–***				
*2. Misuse of pronouns*	*0.44*	***–***			
*3. Invasion of bodily privacy*	*0.46*	*0.38*	***–***		
*4. Behavioural discomfort*	*0.46*	*0.31*	*0.54*	***–***	
*5. Denial of societal transphobia*	*0.56*	*0.41*	*0.48*	*0.49*	***–***

In multivariable models for GIMS subscales, we found evidence to support a positive association between four subscales and specific mental health outcomes: behavioural discomfort from others, denial of societal transphobia, misuse of pronouns, and denial of gender identity, (see Table 4). Findings for each of these subscales were as follows, each with estimates from fully adjusted multivariable models, including mutual adjustment for other subscales Table 5.

**Table 4:** Associations between specific microaggressions (GIMS subscales) and depression and anxiety outcomes

N = 574	Depressive symptoms		Anxiety symptoms	
Unadjusted Models	Coefficient (95%CI)	P-value	Coefficient (95%CI)	P-value
Denial of gender identity	1.58 (1.06 to 2.10)	< 0.001	1.32 (0.83 to 1.80)	< 0.001
Misuse of pronouns	1.18 (0.65 to 1.71)	< 0.001	1.05 (0.55 to 1.54)	< 0.001
Invasion of bodily privacy	1.62 (1.10 to 2.14)	< 0.001	1.39 (0.90 to 1.87)	< 0.001
Behavioural discomfort	1.74 (1.23 to 2.24)	< 0.001	1.31 (0.83 to 1.79)	< 0.001
Denial of societal transphobia	1.74 (1.23 to 2.26)	< 0.001	1.59 (1.11 to 2.07)	< 0.001

Partially Adjusted Models*	Coefficient (95%CI)	P-value	Coefficient (95%CI)	P-value
Denial of gender identity	0.43 (-0.23 to 1.09)	0.205	0.29 (-0.33 to 0.91)	0.359
Misuse of pronouns	0.21 (-0.39 to 0.80)	0.495	0.22 (-0.33 to 0.77)	0.434
Invasion of bodily privacy	0.54 (-0.11 to 1.18)	0.104	0.53 (-0.08 to 1.13)	0.086
Behavioural discomfort	0.82 (0.19 to 1.46)	0.011	0.39 (-0.20 to 0.99)	0.194
Denial of societal transphobia	0.76 (0.10 to 1.42)	0.025	0.89 (0.27 to 1.51)	0.005

Fully Adjusted Models**	Coefficient (95%CI)	P-value	Coefficient (95%CI)	P-value
Denial of gender identity	0.19 (-0.45 to 0.83)	0.558	0.09 (-0.52 to 0.71)	0.764
Misuse of pronouns	0.13 (-0.45 to 0.71)	0.661	0.16 (-0.39 to 0.72)	0.562
Invasion of bodily privacy	0.56 (-0.11 to 1.22)	0.099	0.47 (-0.16 to 1.11)	0.141
Behavioural discomfort	0.97 (0.35 to 1.59)	0.002	0.55 (-0.04 to 1.14)	0.068
Denial of societal transphobia	0.59 (-0.05 to 1.23)	0.071	0.80 (0.19 to 1.41)	0.010

^*^Mutually adjusted for each GIMS subscale

^**^ Mutually adjusted for each GIMS subscale plus covariates agreed a priori (age, perceived gender, ethnicity, sexuality, disability (physical or mental health condition that lasts 12 months or more), education, stage of physical/medical transition, and stage of social transition)

figures in bold are significant at p < 0.05

**Table 5:** Associations between specific microaggressions (GIMS subscales) and lifetime NSSH, suicidal thoughts, and suicide attempt

N = 574	Lifetime suicidal thoughts		Lifetime suicide attempt		Lifetime non-suicidal self-harm	
Unadjusted Models	OR_crude_ (95%CI)	P-value	OR_crude_ (95%CI)	P-value	OR_crude_ (95%CI)	P-value
Denial of gender identity	2.05 (1.48–2.83)	< 0.001	1.66 (1.39–1.99)	< 0.001	1.77 (1.43–2.18)	< 0.001
Misuse of pronouns	1.90 (1.51–2.38)	< 0.001	1.37 (1.14–1.66)	0.001	1.79 (1.49–2.14)	< 0.001
Invasion of bodily privacy	1.84 (1.33–2.53)	< 0.001	1.45 (1.22–1.73)	< 0.001	1.55 (1.26–1.91)	< 0.001
Behavioural discomfort	2.06 (1.41–3.02)	< 0.001	1.32 (1.12–1.56)	0.001	1.48 (1.19–1.84)	< 0.001
Denial of societal transphobia	2.21 (1.58–3.08)	< 0.001	1.41 (1.18–1.67)	< 0.001	1.58 (1.29–1.94)	< 0.001
Partially Adjusted Models*	OR_*adj (*_95%CI)	P-value	OR_*adj*_ (95%CI)	P-value	OR_*adj*_ (95%CI)	P-value
Denial of gender identity	1.18 (0.78–1.78)	0.433	1.48 (1.18–1.85)	0.001	1.32 (1.00–1.73)	0.048
Misuse of pronouns	1.45 (1.10–1.91)	0.009	1.07 (0.86–1.33)	0.526	1.49 (1.20–1.84)	< 0.001
Invasion of bodily privacy	1.05 (0.69–1.58)	0.832	1.19 (0.95–1.48)	0.128	1.11 (0.85–1.45)	0.461
Behavioural discomfort	1.26 (0.79–1.99)	0.334	0.98 (0.79–1.22)	0.879	1.02 (0.77–1.34)	0.888
Denial of societal transphobia	1.48 (0.97–2.28)	0.071	1.04 (0.83–1.30)	0.744	1.08 (0.82–1.42)	0.567
Fully Adjusted Models**	OR_*adj*_ (95%CI)	P-value	OR_*adj*_ (95%CI)	P-value	OR_*adj*_ (95%CI)	P-value
Denial of gender identity	1.10 (0.71–1.70)	0.670	1.40 (1.11–1.77)	0.005	1.28 (0.96–1.71)	0.095
Misuse of pronouns	1.49 (1.09–2.03)	0.013	1.13 (0.90–1.42)	0.280	1.46 (1.175–1.84)	0.002
Invasion of bodily privacy	0.86 (0.55–1.34)	0.513	1.16 (0.91–1.48)	0.220	1.09 (0.81–1.47)	0.559
Behavioural discomfort	1.33 (0.82–2.15)	0.246	0.96 (0.77–1.20)	0.728	1.06 (0.79–1.42)	0.686
Denial of societal transphobia	1.46 (0.93–2.30)	0.103	1.05 (0.83–1.32)	0.702	1.02 (0.76–1.37)	0.879

*OR*_*crude*_ Unadjusted odds ratio

*OR*_*adj*_ Adjusted odds ratio

figures in bold are significant at p < 0.05

* Mutually adjusted for each GIMS subscale

** Mutually adjusted for each GIMS subscale plus covariates agreed a priori (age, perceived gender, ethnicity, sexuality, disability (physical or mental health condition that lasts 12 months or more), education, stage of physical/medical transition, and stage of social transition)

*Behavioural discomfort:* As scores on the behavioural discomfort from others subscale increased by one standard deviation this was associated with an increase in depressive symptoms (adjusted coefficient 0.97 95%CI = 0.35– 1.59).

*Denial of societal transphobia*: As scores on the denial of societal transphobia subscale increased by one standard deviation this was associated with an increase in anxiety symptoms (adjusted coefficient 0.80 95%CI = 0.19– 1.41).

*Misuse of pronouns***:** As scores on the misuse of pronouns subscale increased by one standard deviation this was associated with increased odds of NSSH (OR_crude_ 1.95, 95%CI = 1.57–2.42; OR_adj_ 1.83, 95%CI = 1.45– 2.30), and with an increased odds of suicidal thoughts (OR_adj_ 1.49 95%CI = 1.10– 2.03).

*Denial of gender identity*: As scores on the denial of gender identity subscale increased by one standard deviation, this was associated with increased odds of suicide attempt (OR_adj_ 1.40 95%CI = 1.11– 1.77).

### Sensitivity analyses

Our comparison of samples with complete and missing data (see Supplementary Table 2) identified differences on seven variables. Comparing models before and after imputation, we found similar estimates of the coefficients and odds ratios in the main analysis and in our two imputed models, with some attenuation in the coefficients as sample sizes increased for our imputed models (see Supplementary Table 3 for comparison of main models and MICE models in the main analysis). Similarly, in our exploratory analyses, we observed minimal attenuation when comparing the complete case analysis and the two imputation models (see Supplementary Table 4, 5, and 6 for comparison of main models and MICE models in the hypothesis-generating analyses).

After adding loneliness to our five main fully adjusted models, we found no significant attenuation of any of the associations (supplementary Table 7), providing no evidence to support loneliness as a putative mediator of these associations.

## Discussion

### Main findings

Analysing data from a sample of trans people from across the UK, we found that experiences of microaggressions were common and were associated with increased severity of depressive symptoms and anxiety symptoms, and increased odds of lifetime NSSH, suicidal thoughts, and suicide attempts. This supported our hypothesis that experiencing more microaggressions would be associated with greater mental health symptoms in trans people compared to those who experienced fewer microaggressions, tested for the first time using validated measures. In exploratory analyses we also found evidence to support associations between specific types of microaggression and selected mental health outcomes: behavioural discomfort with depressive symptoms, denial of societal transphobia with anxiety symptoms, misuse of pronouns with NSSH and suicidal thoughts, and denial of gender identity with suicide attempts. These exploratory findings may allow more specific hypotheses as to the role specific microaggression experiences might play in causal pathways to the development of depressive symptoms, anxiety symptoms, NSSH, suicidal ideation, and suicide attempts. Our findings were unchanged when testing the potential biases introduced by missing data.

### Findings in the context of other studies

Our findings are generally consistent with other empirical evidence on microaggressions describing associations with poorer mental health and wellbeing in other minoritised and marginalised communities, such as minority ethnic communities and LGB communities [56, 57]. They are also consistent with evidence supporting an association between microaggressions and general distress and wellbeing measures among trans people [16, 18, 27, 58, 59]. The findings are also in agreement with the only other study of microaggressions and mental health in the UK trans population, namely our finding between denial of societal transphobia and anxiety symptoms [18]. However, this study improves on previous research [27, 58, 59] by using validated measures of gender identity microaggressions and adding to broad findings on general distress and wellbeing [16] by investigating common mental health problems in the general population. The current study has also generated further hypotheses to test regarding specific microaggression experiences and their associations with specific mental health outcomes.

Several mechanisms may explain associations between the specific microaggression experiences and specific mental health outcomes. First, experiencing behavioural discomfort from others may increase depressive symptoms by reinforcing feelings of being socially excluded, which in turn increases social isolation and loneliness [60]. Evidence from general population samples identifies the role of loneliness in the development of depressive symptoms [61]. Experiences of discrimination can compound loneliness. Whilst we found no evidence that loneliness helped explain the association between trans microaggressions and poor mental health, there is a need for longitudinal work in large, representative samples to explore the relationships between these variables, and specifically the role of loneliness as a mediator. Second, denial of societal transphobia fosters an unsafe environment for trans people to express their concerns about transphobic experiences they may have had. This may increase worry and rumination around lived experiences of transphobia, contributing to anxiety[62]. Third, misuse of pronouns may increase NSSH and suicidal thoughts by signaling to a trans person that they do not belong, giving rise to a sense of thwarted belongingness that drives (according to the Interpersonal Theory of Suicide) self-harm and suicidal ideation [63]. Finally, denial of gender identity may single trans people out and also contribute to a sense of thwarted belongingness, thereby increasing the risk of self-harm and suicidal ideation [63].

### Strengths and limitations

Strengths of this study include a sample size that is larger than other studies examining microaggressions using the GIMS (N = 292), the use of a measure of microaggressions psychometrically validated with trans community members, and the use of including validated measures of mental health [16]. We used robust statistical models, with confounders chosen a priori, and used a range of sensitivity analyses to test for the influence of missing data on estimates.

Our sampling method carries a risk of selection bias and digital exclusion due to our snowball sampling methods. Selection bias may have arisen due to selective avoidance and selective sharing of the recruitment call. Our comparison of samples with missing and complete data suggests that those at an earlier stage of transition were more likely to drop out and had higher odds of NSSH, suicidal ideation, and suicide attempts, but lower scores on depressive symptoms and anxiety symptoms. There may have been overrepresentation of those with poor mental health thus inflating the reported prevalence of mental health conditions. However, our estimates of the associations of poor mental health with microaggressions should remain valid [64, 65].

Recent census data on gender diversity within the UK suggests that 16–24 year-olds were the age group most likely to indicate that their gender identity was different to their sex assigned at birth, followed by 25–34 year-olds [3, 4]. This is consistent with the 73% of respondents to our survey who were aged under 35. However, our study has an under-representation from minority ethnic communities when compared to the general population distribution [50]. This under-representation may have been influenced by the socio-demographic composition of the co-production team and the wider research team. We lacked advice from trans people of colour during the design stage and therefore could have omitted to cover issues salient to these communities within our survey, or indeed sampling considerations that would have improved ethnic minority representation. Future research should prioritise diversity within research and co-production teams and make efforts to ensure adequate sampling from minority ethnic communities to improve the diversity and representativeness of the study sample. These might include, for example, appropriate incentivisation and outreach efforts [66].

It is important to recognise the role of intersectionality between ethnicity, disability, and sexuality when considering microaggressions [67]. Multiple intersecting identities increase the risk of exposure to microaggressions, and in turn the risk for mental ill health [67]. Whilst we did collect data on ethnicity, disability, and sexuality we lacked statistical power to investigate the effects of intersectionality. Further work is needed to explore associations within intersecting identities with regards to microaggression experiences and mental health outcomes.

The cross-sectional nature of the study means we were unable to establish temporality in the associations observed. The measures we used to capture microaggression experiences, NSSH, suicidal ideation, and suicide attempts related to lifetime experiences, whereas depressive symptoms and anxiety symptoms were assessed over the previous two weeks. With data collection at one time point, we were unable to establish whether the exposure (microaggression experiences) preceded the symptoms. We therefore cannot rule out reverse causality in the association between microaggressions and mental health outcomes. There is potential for a bi-directional relationship between microaggressions and mental health, [68]. Longitudinal studies are needed to examine the directionality of these associations. Furthermore, as with any observational study, we are unable to rule out any residual confounding from unmeasured variables or confounders measured imperfectly (such as access to gender-affirming care), which may partially explain the associations observed in this study. We adjusted our final models for stage of physical transition and stage of social transition but ideally would have liked to have collected primary data on access to gender-affirming care. Similarly, our sensitivity analyses tested whether there was evidence to support social support as a mediator of these associations, by adding loneliness to final models. This was an inadequate proxy for poor social support, and ideally, we would have liked to conduct formal mediation analysis using longitudinal data and a validated measure of social support.

### Implications

Public policy and education could reduce the occurrence of microaggressions. Research to develop our understanding of why microaggressions are enacted and how best to reduce their occurrence would lead to better interventions, and furthermore to better mental health outcomes for trans people. Interventions developed for ethnic minority communities include workshops that include the targeted minority alongside the wider community, and other interventions that promote social connectedness, familiarity, closeness, and management of uncomfortable feelings, and reduce social distance [69]. Findings suggest that white students randomized to a Racial Harmony Workshop were less likely to perpetrate microaggressions towards minority ethnic students [69]. However, as highlighted in critiques of the literature on the effectiveness of interventions to address microaggressions, we need more research on effectiveness and acceptability [70]. Given our study’s findings relating to GIMS subscales, a better understanding of how specific microaggression types influence specific mental health outcomes would also allow clinicians to tailor support around how these microaggressions interact with psychiatric presentations in trans people.

Further research is needed to strengthen our understanding on whether microaggressions cause changes in depressive symptoms, anxiety symptoms, NSSH, suicidal ideation, and suicide attempt. Longitudinal studies are needed to answer questions about whether experiencing microaggressions increases the risk of subsequent depressive and anxiety symptoms, NSSH, suicidal ideation, and suicide attempts. Understanding the temporal relationship between microaggressions and mental health using longitudinal designs will help researchers and clinicians better understand both the short and long-term associations of microaggressions with mental health, NSSH, suicidal ideation, and suicide attempts, and any reciprocal influences, identify mediators, and tailor intervention design around these findings. Given the complexity of microaggression experiences, we also recommend further qualitative work through focus groups and interviews to understand how each subscale may be experienced in relation to specific mental health outcomes. Qualitative data regarding microaggression experiences and depressive symptoms, anxiety symptoms, suicidal ideation, and suicide attempts will provide a more nuanced understandings of mechanisms underlying the associations described here.

## Supplementary Information

Below is the link to the electronic supplementary material.

Supplementary file1 (DOCX 61 KB)

## Data Availability

Anonymised data will be made available through the UK Data Service upon publication of these study findings.
