# Supplementary material for: The association between microaggressions and mental health among UK trans people: a cross-sectional study
Source: Soc Psychiatry Psychiatr Epidemiol. 2024 Sep 26;60(5):1211–25. doi: 10.1007/s00127-024-02775-2 (PMC12119694; doi:10.1007/s00127-024-02775-2)
Supplement: Supplementary file 1 — Supplementary file1 (DOCX 61 KB) [file 127_2024_2775_MOESM1_ESM.docx]

Supplementary Materials

**[Supplementary Methods](#_Toc175651265)** [1](#_Toc175651265)

[sTable 3: Findings from multiple imputation analysis of GIMS and depressive symptoms, anxiety symptoms, suicidality and NSSH 4](#_Toc175651266)

[sTable 4: Multiply imputed multivariable models of GIMS subscales with mental health outcomes 5](#_Toc175651267)

[sTable 5: Multiply imputed multivariable logistic regression restricted to complete cases on exposure (GIMS) 6](#_Toc175651268)

[sTable6: Multiply imputed multivariable logistic regression models, unadjusted and adjusted, for the GIMS subscales and suicidality and NSSH outcomes containing imputed data on exposure, confounders, and outcomes, and not restricted to complete cases. 7](#_Toc175651269)

[sTable 7: Associations between microaggressions (total GIMS score) and depressive symptoms, anxiety symptoms, suicidality, and non-suicidal self-harm with final model additionally adjusted for loneliness. 8](#_Toc175651270)

# **Supplementary Methods**

**Sample size**

In the absence of published effect sizes in the literature, we conducted several *a priori* sample size calculations using estimated effect sizes that incrementally increased by 0.05 up to a beta coefficient of 0.50. Assuming a conservative effect size of 0.15, we estimated that a sample of 463 participants was required for 90% power at an alpha level of 0.05.

sTable 1: Full list of the 14 items in the five sub-scales of the Gender Identity Microaggression Scale (GIMS)

|  | 1 | 2 | 3 | 4 | 5 |
| --- | --- | --- | --- | --- | --- |
| **Subscale 1: Denial of Gender Identity** |  |  |  |  |  |
| A loved one (e.g., family or friend) told me that my gender nonconformity is just a phase |  |  |  |  |  |
| Someone told me that my transgender identity or my gender nonconformity was just a phase |  |  |  |  |  |
| I was told that I made a family member uncomfortable because of my gender nonconformity or transgender identity |  |  |  |  |  |
| LGB people have told me that my gender nonconformity is just a phase |  |  |  |  |  |
| **Subscale 2: Misuse of Pronouns** |  |  |  |  |  |
| Strangers and acquaintances have called me by the wrong personal pronoun |  |  |  |  |  |
| A loved one (e.g., family or friend) has called me by the wrong personal pronoun |  |  |  |  |  |
| **Subscale 3: Invasion of Bodily Privacy** |  |  |  |  |  |
| Someone wanted to engage in a sexual act with me only because they view transgender people as exotic |  |  |  |  |  |
| Someone (e.g., family, friend, co-worker) has asked me personal questions about gender reassignment… |  |  |  |  |  |
| Someone (e.g., family, friend, co-worker) has asked me if I feel I’m trapped in the wrong body |  |  |  |  |  |
| **Subscale 4: Behavioural Discomfort from Others** |  |  |  |  |  |
| Someone avoided sitting next to me in a public or government setting (e.g., DMV, courthouses, libraries) |  |  |  |  |  |
| Someone avoided sitting next to me at a bar or restaurant because I am gender nonconforming |  |  |  |  |  |
| My employer or co-worker was unfriendly to me because I dress gender nonconforming |  |  |  |  |  |
| **Subscale 5: Denial of Societal Transphobia** |  |  |  |  |  |
| I was told that I complain too much about societal discrimination against gender nonconforming people |  |  |  |  |  |
| I was told that I complain too much about how people react to my gender nonconformity |  |  |  |  |  |

sTable 2: Comparison of samples with missing and complete data on microaggressions

|  | **Overall sample** | | |
| --- | --- | --- | --- |
| **Variable** | Missing (%) | Complete (%) | P-value |
| **Gender Identity**  Trans men  Trans women  Nonbinary | 33 (24.1%) 46 (19.7%) 89 (26.2%) | 104 (75.9%) 188 (80.3%) 251 (73.8%) | 0.194 |
| **Currently living in affirmed gender**  Yes, either all or most of the time  No, not living in affirmed gender | 145 (23.4%) 33 (25.2%) | 476 (76.6%) 98 (74.8%) | 0.652 |
| **Perceived gender by others**  As a trans person  As the sex assigned at birth  As the gender identified  Does not know  Other | 26 (18.6%) 35 (23.2%) 83 (25.2%) 16 (24.6%) 18 (27.3%) | 114 (81.4%) 116 (76.8%) 247 (74.8%) 49 (75.4%) 48 (72.7%) | 0.568 |
| **Physical transition**  No, has not undergone/not relevant  Yes, proposing to undergo  Yes, currently undergoing  Yes, undergone  Unsure/Prefer not to say/Other | 28 (28.6%) 36 (22.6%) 61 (23.4%) 27 (19.3%) 26 (27.7%) | 70 (71.4%) 123 (77.4%) 200 (76.6%) 113 (80.7%) 68 (72.3%) | 0.444 |
| **Social transition**  No, has not undergone/not relevant  Yes, proposing to undergo  Yes, currently undergoing  Yes, undergone  Unsure/Prefer not to say/Other | **8 (38.1%)** **11 (20.0%)** **55 (25.5%)** **87 (20.6%)** **17 (44.7%)** | **13 (61.9%)** **44 (80.0%)** **161 (74.5%)** **335 (79.4%)** **21 (55.3%)** | **0.005** |
| **Age (years)**  18-25  26-34  35-44  45+ | 76 (25.3%) 71 (26.9%) 28 (24.4%) 25 (26.6%) | 225 (74.7%) 193 (73.1%) 87 (75.6%) 69 (73.4%) | 0.946 |
| **Country of current residence**  England  Northern Ireland  Scotland  Wales | 124 (20.4%) <5 (40.0%) 12 (15.4%) <5 (17.4%) | 483 (79.6%) 6 (60.0%) 66 (84.6%) 19 (82.6%) | 0.296 |
| **Urbanicity**  Urban  Rural  Do not know/Other | **122 (22.4%)** **20 (13.1%)** **<5 (18.2%)** | **423 (77.6%)** **133 (86.9%)** **18 (81.8%)** | **0.039** |
| **Ethnicity**  Ethnic minority  White | 6 (10.9%) 27 (4.9%) | 525 (89.1%) 49 (95.1%) | 0.061 |
| **Education**  No formal education  GCSEs or equivalent  A’ Levels, Scottish Highers  University Degree, e.g., BSc, BA  Master’s Degree or equivalent  Doctorate, e.g., MD, PhD  Vocational qualifications | 4 (26.7%) 5 (12.5%) 29 (20.1%) 43 (18.2%) 43 (24.2%) 13 (25.0%) 9 (16.4%) | 11 (73.3%) 35 (87.5%) 115 (79.9%) 193 (81.8%) 135 (75.8%) 39 (75.0%) 46 (83.6%) | 0.495 |
| **Sexuality**  LGB/Queer/Questioning/Asexual/Aromantic  Heterosexual | 131 (19.3%) 12 (31.6%) | 548 (80.7%) 26 (68.4%) | 0.065 |
| **Disability expected to last 12 months or longer**  No  Yes | 36 (20.7%) 107 (19.7%) | 138 (79.3%) 436 (80.3%) | 0.777 |
| **Disability reducing ability to carry out day to day activities**  Not at all  Yes, a little  Yes, a lot | 7 (21.2%) 69 (20.2%) 28 (17.0%) | 26 (78.8%) 273 (79.8%) 137 (83.0%) | 0.663 |
| **Diagnosed mental health condition**  No  Yes  Prefer not to say/do not know | 9 (25.7%)  116 (18.5%)  9 (19.6%) | 26 (74.3%) 511 (81.5%) 37 (80.4%) | 0.675 |
| **PHQ-9 (depressive symptoms, past two weeks)** | **9.84 (6.81)** | **12.06 (6.49)** | **0.001** |
| **GAD-7 (anxiety symptoms, past two weeks)** | **8.75 (6.32)** | **10.07 (6.02)** | **0.03** |
| **Lifetime history of thinking life is not worth living (passive death wish)**  No  Yes | **20 (30.3%)**  **144 (17.8%)** | **46 (69.7%)** **528 (82.2%)** | **0.013** |
| **Lifetime history of wishing one were dead (passive death wish)**  No  Yes | **28 (31.5%)** **106 (17.1%)** | **61 (68.5%)** **513 (82.9%)** | **0.001** |
| **Lifetime history of suicidal thoughts**   No  Yes | **24 (32.9%)** **110 (17.3%)** | **49 (67.1%)** **525 (82.7%)** | **0.001** |
| **Lifetime** **history of suicide attempts**   No  Yes | 89 (20.6%) 45 (16.3%) | 343 (79.4%) 231 (83.7%) | 0.155 |
| **Lifetime history of non-suicidal self-harm**   No  Yes | **42 (25.0%)** **92 (17.0%)** | **126 (75.0%)** **448 (83.0%)** | **0.021** |

PHQ-9 – Patient Health Questionnaire – 9 item version
GAD-7 – Generalised Anxiety Disorder scale – 7 item version
Figures in bold are significant at p<0.05

## sTable 3: Findings from multiple imputation analysis of GIMS and depressive symptoms, anxiety symptoms, suicidality and NSSH

|  | *Gender Identity Microaggressions (GIMS)*    *Model one†* | | | | *Gender Identity Microaggressions (GIMS)*    *Model two††* | | | |
| --- | --- | --- | --- | --- | --- | --- | --- | --- |
|  | *Unadjusted* | | *Adjusted†††* | | *Unadjusted* | | *Adjusted†††* | |
|  | Model N | Coefficient (95%CI) | Model N | Coefficient (95%CI) | Model N | Coefficient (95%CI) | Model N | Coefficient (95%CI) |
| PHQ-9 – Depressive symptoms (past two weeks) | 679 | **2.09* (1.62 to 2.57)** | 679 | **1.72* (1.24 to 2.20)** | 787 | **1.88* (1.40 to 2.36)** | 787 | **1.49* (1.01 to 1.97)** |
| GAD-7 – Anxiety symptoms (past two weeks) | 679 | **1.69* (1.24 to 2.13)** | 679 | **1.41* (0.95 to 1.86)** | 787 | **1.51* (1.06 to 1.96)** | 787 | **1.22* (0.73 to 1.70)** |
|  | Model N | OR_crude_ (95%CI) | Model N | OR*_adj_* (95%CI) | Model N | OR_crude_ (95%CI) | Model N | OR*_adj_* (95%CI) |
| Lifetime suicidal thoughts  No  Yes | 679 | **1**  **2.57* (1.93 to 3.43)** | 679 | **1**  **2.09* (1.53 to 2.87)** | 787 | **1**  **2.27* (1.71 to 3.01)** | 787 | **1**  **1.87* (1.39 to 2.52)** |
| Lifetime suicide attempt  No  Yes | 679 | **1**  **1.68* (1.42 to 1.99)** | 679 | **1**  **1.57* (1.31 to 1.87)** | 787 | **1**  **1.60* (1.35 to 1.89)** | 787 | **1**  **1.48* (1.25 to 1.76)** |
| Lifetime non-suicidal self-harm  No  Yes | 679 | **1**  **1.85* (1.53 to 2.24)** | 679 | **1**  **1.71* (1.38 to 2.10)** | 787 | **1**  **1.74* (1.43 to 2.10)** | 787 | **1**  **1.61* (1.31 to 1.99)** |

† Model one contains imputed confounders and outcomes and is restricted to complete cases on exposure (GIMS)
†† Model two contains imputed exposure, confounders, and outcomes, and is not restricted to complete cases
††† Adjusted for age, perceived gender, ethnicity, sexuality, disability, education, stage of physical/medical transition, and stage of social transition
* figures in bold are significant at p<0.001
PHQ – Patient Health Questionnaire
GAD – Generalised Anxiety Disorder Scale

## sTable 4: Multiply imputed multivariable models of GIMS subscales with mental health outcomes

|  | Depressive symptoms (past two weeks) | | | | Anxiety symptoms (past two weeks) | | | |
| --- | --- | --- | --- | --- | --- | --- | --- | --- |
|  | ***Model one† (N=679)*** | | *Model two†† (N=787)* | | *Model one† (N=679)* | | *Model two†† (N=787)* | |
| **Unadjusted**  **Models** | Mean difference (95%CI) | P-value | Mean difference (95%CI) | P-value | Mean difference (95%CI) | P-value | Mean difference (95%CI) | P-value |
| Denial of gender identity | **1.53 (1.04 to 2.01)** | **<0.001** | **1.54 (1.07 to 2.02)** | **<0.001** | **1.26 (0.81 to 1.71)** | **<0.001** | **1.26 (0.79 to 1.73)** | **<0.001** |
| Misuse of pronouns | **1.13 (0.64 to 1.62)** | **<0.001** | **1.11 (0.62 to 1.60)** | **<0.001** | **1.02 (0.57 to 1.48)** | **<0.001** | **1.00 (0.54 to 1.46)** | **<0.001** |
| Invasion of bodily privacy | **1.65 (1.17 to 2.13)** | **<0.001** | **1.65 (1.17 to 2.14)** | **<0.001** | **1.30 (0.85 to 1.75)** | **<0.001** | **1.29 (0.81 to 1.77)** | **<0.001** |
| Behavioural discomfort | **1.76 (1.28 to 2.24)** | **<0.001** | **1.76 (1.27 to 2.24)** | **<0.001** | **1.25 (0.80 to 1.70)** | **<0.001** | **1.26 (0.78 to 1.73)** | **<0.001** |
| Denial of societal transphobia | **1.76 (1.28 to 2.23)** | **<0.001** | **1.75 (1.27 to 2.23)** | **<0.001** | **1.54 (1.09 to 1.98)** | **<0.001** | **1.53 (1.06 to 2.00)** | **<0.001** |
| **Adjusted**  **Models*** | Mean difference (95%CI) | P-value | Mean difference (95%CI) | P-value | Mean difference (95%CI) | P-value | Mean difference (95%CI) | P-value |
| Denial of gender identity | 0.34 (-0.28 to 0.96) | 0.277 | 0.37 (-0.26 to 1.00) | 0.245 | 0.26 (-0.32 to 0.84) | 0.374 | 0.28 (-0.31 to 0.87) | 0.357 |
| Misuse of pronouns | 0.16 (-0.39 to 0.71) | 0.565 | 0.14 (-0.41 to 0.70) | 0.618 | 0.25 (-0.26 to 0.77) | 0.329 | 0.23 (-0.29 to 0.76) | 0.384 |
| Invasion of bodily privacy | **0.60 (0.00 to 1.20)** | **0.050** | 0.61 (0.00 to 0.22) | 0.051 | 0.46 (-0.10 to 1.03) | 0.107 | 0.45 (-0.12 to 1.02) | 0.121 |
| Behavioural discomfort | **0.84 (0.24 to 1.43)** | **0.006** | **0.83 (0.22 to 1.44)** | **0.008** | 0.36 (-0.20 to 0.92) | 0.208 | 0.38 (-.19 to 0.95) | 0.195 |
| Denial of societal transphobia | **0.81 (0.19 to 1.43)** | **0.010** | **0.79 (0.13 to 1.46)** | **0.020** | **0.90 (0.32 to 1.47)** | **0.002** | **0.89 (0.28 to 1.50)** | **0.004** |
| **Adjusted**  **Models**** | Mean difference (95%CI) | P-value | Mean difference (95%CI) | P-value | Mean difference (95%CI) | P-value | Mean difference (95%CI) | P-value |
| Denial of gender identity | 0.18 (-0.43 to 0.78) | 0.565 | 0.14 (-0.48 to 0.76) | 0.650 | 0.14 (-0.43 to 0.71) | 0.634 | 0.09 (-0.49 to 0.68) | 0.754 |
| Misuse of pronouns | -0.12 (-0.68 to 0.43) | 0.668 | -0.11 (-0.66 to 0.44) | 0.694 | -0.03 (-0.56 to 0.50) | 0.915 | -0.02 (-0.55 to 0.52) | 0.955 |
| Invasion of bodily privacy | 0.55 (-0.07 to 1.17) | 0.081 | 0.54 (-0.09 to 1.17) | 0.091 | 0.43 (-0.15 to 1.02) | 0.148 | 0.41 (-0.19 to 1.01) | 0.183 |
| Behavioural discomfort | **0.95 (0.36 to 1.53)** | **0.002** | **0.94 (0.37 to 1.51)** | **0.001** | 0.51 (-0.05 to 1.06) | 0.075 | 0.51 (-0.04 to 1.06) | 0.066 |
| Denial of societal transphobia | **0.66 (0.06 to 1.25)** | **0.031** | **0.64 (0.01 to 1.27)** | **0.046** | **0.77 (0.20 to 1.33)** | **0.008** | **0.76 (0.15 to 1.37)** | **0.015** |

† Model one contains imputed confounders and outcomes and is restricted to complete cases on exposure (GIMS)
†† Model two contains imputed exposure, confounders, and outcomes, and is not restricted to complete cases
*Mutually adjusted for each GIMS subscale
** Mutually adjusted for each GIMS subscale plus age, perceived gender, ethnicity, sexuality, disability, education, stage of physical/medical transition, and stage of social transition
Figures in bold are significant at p<0.05

## sTable 5: Multiply imputed multivariable logistic regression restricted to complete cases on exposure (GIMS)

| Model one (N=679) | Lifetime suicidal thoughts | | Lifetime suicide attempts | | Lifetime non-suicidal self-harm | |
| --- | --- | --- | --- | --- | --- | --- |
| **Unadjusted**  **Models** | OR_crude_ (95%CI) | P-value | OR_crude_ (95%CI) | P-value | OR_crude_ (95%CI) | P-value |
| Denial of gender identity | **1.98 (1.51 to 2.62)** | **<0.001** | **1.62 (1.38 to 1.91)** | **<0.001** | **1.64 (1.36 to 1.98)** | **<0.001** |
| Misuse of pronouns | **1.84 (1.50 to 2.25)** | **<0.001** | **1.32 (1.12 to 1.57)** | **0.001** | **1.67 (1.42 to 1.97)** | **<0.001** |
| Invasion of bodily privacy | **1.91 (1.44 to 2.53)** | **<0.001** | **1.48 (1.26 to 1.74)** | **<0.001** | **1.53 (1.27 to 1.84)** | **<0.001** |
| Behavioural discomfort | **2.02 (1.45 to 2.80)** | **<0.001** | **1.38 (1.18 to 1.61)** | **<0.001** | **1.45 (1.19 to 1.76)** | **<0.001** |
| Denial of societal transphobia | **2.34 (1.74 to 3.14)** | **<0.001** | **1.45 (1.24 to 1.70)** | **<0.001** | **1.60 (1.33 to 1.92)** | **<0.001** |
| **Partially Adjusted**  **Models*** | OR*_adj_* (95%CI) | P-value | OR*_adj_* (95%CI) | P-value | OR*_adj_* (95%CI) | P-value |
| Denial of gender identity | 1.11 (0.78 to 1.58) | 0.549 | **1.39 (1.13 to 1.71)** | **0.002** | 1.20 (0.94 to 1.53) | 0.145 |
| Misuse of pronouns | **1.38 (1.08 to 1.76)** | **0.010** | 1.02 (0.84 to 1.24) | 0.848 | **1.39 (1.15 to 1.69)** | **0.001** |
| Invasion of bodily privacy | 1.14 (0.80 to 1.62) | 0.481 | 1.20 (0.98 to 1.47) | 0.078 | 1.12 (0.88 to 1.42) | 0.352 |
| Behavioural discomfort | 1.16 (0.78 to 1.73) | 0.456 | 1.04 (0.85 to 1.26) | 0.719 | 1.02 (0.80 to 1.31) | 0.873 |
| Denial of societal transphobia | **1.66 (1.14 to 2.41)** | **0.008** | 1.09 (0.89 to 1.34) | 0.388 | 1.18 (0.92 to 1.50) | 0.191 |
| **Fully Adjusted**  **Models**** | OR*_adj_* (95%CI) | P-value | OR*_adj_* (95%CI) | P-value | OR*_adj_* (95%CI) | P-value |
| Denial of gender identity | 1.14 (0.78 to 1.67) | 0.489 | **1.37 (1.10 to 1.70)** | **0.005** | 1.19 (0.92 to 1.54) | 0.197 |
| Misuse of pronouns | 1.26 (0.95 to 1.67) | 0.108 | 1.01 (0.82 to 1.25) | 0.908 | **1.25 (1.01 to 1.23)** | **0.043** |
| Invasion of bodily privacy | 0.94 (0.64 to 1.39) | 0.764 | 1.15 (0.92 to 1.44) | 0.213 | 1.18 (0.90 to 1.54) | 0.226 |
| Behavioural discomfort | 1.25 (0.82 to 1.90) | 0.300 | 1.00 (0.81 to 1.24) | 0.976 | 1.09 (0.84 to 1.42) | 0.523 |
| Denial of societal transphobia | **1.54 (1.03 to 2.29)** | **0.034** | 1.10 (0.89 to 1.36) | 0.382 | 1.07 (0.82 to 1.39) | 0.609 |

*Mutually adjusted for each GIMS subscale
** Mutually adjusted for each GIMS subscale plus age, perceived gender, ethnicity, sexuality, disability, education, stage of physical/medical transition, and stage of social transition
OR_crude_ – Unadjusted Odds Ratio
OR*_adj_* – Adjusted Odds Ratio
Figures in bold are significant at p<0.05

## sTable6: Multiply imputed multivariable logistic regression models, unadjusted and adjusted, for the GIMS subscales and suicidality and NSSH outcomes containing imputed data on exposure, confounders, and outcomes, and not restricted to complete cases.

| Model two (N=787) | Lifetime suicidal thoughts | | Lifetime suicide attempts | | Lifetime non-suicidal self-harm | |
| --- | --- | --- | --- | --- | --- | --- |
| **Unadjusted Models** | **OR_crude_ (95%CI)** | **P-value** | **OR_crude_ (95%CI)** | **P-value** | **OR_crude_ (95%CI)** | **P-value** |
| Denial of gender identity | **1.99 (1.50 to 2.62)** | **<0.001** | **1.61 (1.38 to 1.89)** | **<0.001** | **1.60 (1.33 to 1.92)** | **<0.001** |
| Misuse of pronouns | **1.89 (1.55 to 2.30)** | **<0.001** | **1.30 (1.10 to 1.54)** | **0.002** | **1.68 (1.42 to 2.00)** | **<0.001** |
| Invasion of bodily privacy | **1.82 (1.36 to 2.43)** | **<0.001** | **1.49 (1.27 to 1.74)** | **<0.001** | **1.50 (1.25 to 1.80)** | **<0.001** |
| Behavioural discomfort | **1.86 (1.36 to 2.54)** | **<0.001** | **1.38 (1.18 to 1.61)** | **<0.001** | **1.42 (1.18 to 1.72)** | **<0.001** |
| Denial of societal transphobia | **2.29 (1.73 to 3.02)** | **<0.001** | **1.44 (1.23 to 1.69)** | **<0.001** | **1.58 (1.31 to 1.90)** | **<0.001** |
| **Partially Adjusted**  **Models*** | OR*_adj_* (95%CI) | P-value | OR*_adj_* (95%CI) | P-value | OR*_adj_* (95%CI) | P-value |
| Denial of gender identity | 1.15 (10.79 to 1.67) | 0.474 | **1.39 (1.13 to 1.71)** | **0.002** | 1.17 (0.92 to 1.48) | 0.199 |
| Misuse of pronouns | **1.45 (1.14 to 1.83)** | **0.002** | 1.01 (0.83 to 1.23) | 0.948 | **1.43 (1.17 to 1.74)** | **<0.001** |
| Invasion of bodily privacy | 1.09 (0.75 to 1.56) | 0.658 | 1.22 (0.99 to 1.49) | 0.060 | 1.11 (0.88 to 1.40) | 0.396 |
| Behavioural discomfort | 1.10 (0.74 to 1.64) | 0.626 | 1.03 (0.85 to 1.26) | 0.733 | 1.02 (0.81 to 1.30) | 0.852 |
| Denial of societal transphobia | **1.64 (1.15 to 2.36)** | **0.007** | 1.09 (0.88 to 1.35) | 0.443 | 1.18 (0.92 to 1.51) | 0.201 |
| **Fully Adjusted**  **Models**** | OR*_adj_* (95%CI) | P-value | OR*_adj_* (95%CI) | P-value | OR*_adj_* (95%CI) | P-value |
| Denial of gender identity | 1.16 (0.80 to 1.66) | 0.435 | **1.36 (1.08 to 1.70)** | **0.008** | 1.17 (0.91 to 1.51) | 0.228 |
| Misuse of pronouns | 1.30 (0.98 to 1.73) | 0.071 | 0.99 (0.80 to 1.22) | 0.925 | **1.29 (1.03 to 1.60)** | **0.023** |
| Invasion of bodily privacy | 0.95 (0.64 to 1.41) | 0.809 | 1.16 (0.93 to 1.44) | 0.198 | 1.16 (0.90 to 1.51) | 0.252 |
| Behavioural discomfort | 1.13 (0.77 to 1.67) | 0.540 | 1.00 (0.81 to 1.22) | 0.970 | 1.09 (0.82 to 1.43) | 0.554 |
| Denial of societal transphobia | **1.56 (1.05 to 2.30)** | **0.026** | 1.10 (0.88 to 1.37) | 0.406 | 1.09 (0.84 to 1.42) | 0.525 |

*Mutually adjusted for each GIMS subscale
 ** Mutually adjusted for each GIMS subscale plus age, perceived gender, ethnicity, sexuality, disability, education, stage of physical/medical transition, and stage of social transition
OR_crude_ – Unadjusted Odds Ratio
OR*_adj_* – Adjusted Odds Ratio
 figures in bold are significant at p<0.05

## sTable 7: Associations between microaggressions (total GIMS score) and depressive symptoms, anxiety symptoms, suicidality, and non-suicidal self-harm with final model additionally adjusted for loneliness.

|  | ***Unadjusted*** | | | ***Adjusted**** | | | ***Fully Adjusted***** | | |
| --- | --- | --- | --- | --- | --- | --- | --- | --- | --- |
|  | **Model N** | **Coefficient (95%CI)** | **P-value** | **Model N** | **Coefficient (95%CI)** | **P-value** | **Model N** | **Coefficient (95%CI)** | **P-value** |
| *PHQ-9 – Depressive symptoms*  *Past two weeks* | 511 | 1.96 (1.42 to 2.50) | <0.001 | 511 | 1.71 (1.17 to 2.25) | <0.001 | 511 | 1.30 (0.79 to 1.80) | <0.001 |
| *GAD-7 – Anxiety symptoms*  *Past two weeks* | 511 | 1.59 (1.09 to 2.09) | <0.001 | 511 | 1.41 (0.90 to 1.92) | <0.001 | 511 | 1.04 (0.55 to 1.53) | <0.001 |
|  | **Model N** | **OR_crude_ (95%CI)** | **P-value** | **Model N** | **OR_crude_ (95%CI)** | **P-value** | **Model N** | **OR_adj_ (95%CI)** | **P-value** |
| Lifetime suicidal thoughts    No    Yes | 511 | 2.87 (1.95 to 4.21) | <0.001 | 511 | 2.48 (1.62 to 3.78) | <0.001 | 511 | 2.34 (1.52 to 3.61) | <0.001 |
| Lifetime suicide attempt    No    Yes | 511 | 1.73 (1.43 to 2.10) | <0.001 | 511 | 1.69 (1.37 to 2.08) | <0.001 | 511 | 1.64 (1.33 to 2.02) | <0.001 |
| Lifetime non-suicidal self-harm    No    Yes | 511 | 1.85 (1.48 to 2.33) | <0.001 | 511 | 1.68 (1.32 to 2.15) | <0.001 | 511 | 1.65 (1.29 to 2.11) | <0.001 |

* Linear and logistic regression models were fully adjusted for baseline mental health outcomes as well as perceived gender, sexuality, ethnicity, age, sexuality, stage of physical and/or social transition, and disability 
** Linear and logistic regression models fully adjusted for confounders plus the addition of loneliness 
PHQ – Patient Health Questionnaire  
GAD – Generalised Anxiety Disorder Scale 
GIMS – Gender Identity Microaggressions Scale
